# The predictive value of total-body PET/CT in non-small cell lung cancer for the PD-L1 high expression

**DOI:** 10.3389/fonc.2022.943933

**Published:** 2022-09-23

**Authors:** Bingxin Hu, Huibin Jin, Xiali Li, Xinyu Wu, Junling Xu, Yongju Gao

**Affiliations:** Department of Nuclear Medicine, Henan Key Laboratory of Novel Molecular Probes and Clinical Translation in Nuclear Medicine, Henan Provincial People’s Hospital and the People’s Hospital of Zhengzhou University, Zhengzhou, China

**Keywords:** NSCLC, PET/CT, SUR, PD-L1 (22C3), tumor cells, immune cells

## Abstract

**Purpose:**

Total-body positron emission tomography/computed tomography (PET/CT) provides faster scanning speed, higher image quality, and lower injected dose. To compensate for the shortcomings of the maximum standard uptake value (SUVmax), we aimed to normalize the values of PET parameters using liver and blood pool SUV (SUR-L and SUR-BP) to predict programmed cell death-ligand 1 (PD-L1) expression in non-small cell lung cancer (NSCLC) patients.

**Materials and methods:**

A total of 138 (104 adenocarcinoma and 34 squamous cell carcinoma) primary diagnosed NSCLC patients who underwent ^18^F-FDG-PET/CT imaging were analyzed retrospectively. Immunohistochemistry (IHC) analysis was performed for PD-L1 expression on tumor cells and tumor-infiltrating immune cells with 22C3 antibody. Positive PD-L1 expression was defined as tumor cells no less than 50% or tumor-infiltrating immune cells no less than 10%. The relationships between PD-L1 expression and PET parameters (SUVmax, SUR-L, and SUR-BP) and clinical variables were analyzed. Statistical analysis included χ^2^ test, receiver operating characteristic (ROC), and binary logistic regression.

**Results:**

There were 36 patients (26%) expressing PD-L1 positively. Gender, smoking history, Ki-67, and histologic subtype were related factors. SUVmax, SUR-L, and SUR-BP were significantly higher in the positive subset than those in the negative subset. Among them, the area under the curve (AUC) of SUR-L on the ROC curve was the biggest one. In NSCLC patients, the best cutoff value of SUR-L for PD-L1-positive expression was 4.84 (AUC = 0.702, P = 0.000, sensitivity = 83.3%, specificity = 54.9%). Multivariate analysis confirmed that age and SUR-L were correlated factors in adenocarcinoma (ADC) patients.

**Conclusion:**

SUVmax, SUR-L, and SUR-BP had utility in predicting PD-L1 high expression, and SUR-L was the most reliable parameter. PET/CT can offer reference to screen patients for first-line atezolizumab therapy.

## Introduction

Lung cancer is the leading cause of cancer morbidity and mortality worldwide (1), and non-small cell lung cancer (NSCLC) contributes approximately 85% (2). Since 2012, clinical studies have confirmed that immune checkpoint inhibitors (ICIs) with programmed death-1 (PD-1) or its ligand [programmed cell death-ligand 1 (PD-L1)] blockade can significantly prolong the survival time compared to traditional therapies in NSCLC (3–7). PD-L1 expression is regulated by two mechanisms: intrinsic expression on tumor cells (TCs) and adaptive expression on tumor-infiltrating immune cells (ICs) (8, 9). According to the Food and Drug Administration (FDA) and European Medicines Agency (EMA), PD-L1 high expression with the expression on TCs no less than 50% or ICs no less than 10% is one of the basis to choose ICIs as the first line in advanced NSCLC patients (10, 11).

Two-deoxy-2-[fluorine-18] fluoro-D-glucose positron emission tomography/computed tomography (^18^F-FDG PET/CT) plays a key role in tumor diagnosis, staging, restaging, and so on and offers important information on clinical treatment options (12, 13). Recently, the total-body PET/CT, uEXPLORER (United Imaging Healthcare, Shanghai, China) with the 194-cm-long Field of View (FOV), has ultrahigh system sensitivity and spatial resolution. Thus, it dramatically improves image quality and ability to detect small lesions and distant metastases (14, 15). Regarding semiquantitative parameters, several studies have found that the standard uptake value ratio (SUR) is a better predictive index than tumor maximum standard uptake value (SUVmax) (16–19). SUR is a value normalized by liver or blood pool standard uptake value (SUV) (20, 21), which can be more stable and reliable.

Although the SUVmax has been described as a predictor of PD-L1 expression and response to immunotherapy in NSCLC patients, its clinical significance remains unclear. Previous research has demonstrated that SUVmax is positively correlated with PD-L1 expression on TCs (22–25). However, that research rarely contains SUR and mainly concentrated on the expression on TCs. Based on all of them, we conducted a retrospective analysis to explore the relationship between PD-L1 high expression and PET parameters that were obtained by the newest PET/CT machine in NSCLC.

## Materials and methods

### Patients

In this study, we enrolled 138 patients with primary NSCLC from June 2020 to December 2021 at the nuclear medicine department of Henan Provincial People’s Hospital, Zhengzhou University. The inclusion criteria were as follows: 1) first diagnosis of NSCLC; 2) integrity of pathological data, including Ki-67, histological subtype, PD-L1 expression on TCs and ICs; 3) total-body PET images before treatments and biopsy. The exclusion criteria were as follows: 1) patients with other tumors or therapy before PET image and immunohistochemistry (IHC); 2) patients with unknown histological subtype; 3) patients with incomplete clinical data, which included age, gender, maximum diameter, smoking history, tumor–node–metastasis stage (Eighth Edition of the Lung Cancer Staging System), and the source of histologic samples. This study protocol was approved by the institutional review board, and the need for a written informed consent was waived (2020 IRB 93th).

### ^18^F-FDG PET/CT

All patients fasted at least 6 h, and serum glucose levels were less than 10 mmol/L before intravenous injection of ^18^F-FDG. After injection, all patients rested approximately 40–60 min and then underwent PET/CT imaging. All images were acquired on total-body PET/CT (uEXPLORER, United Imaging Healthcare, Shanghai, China). The ^18^F-FDG was made by our chemists, and its radiochemical purity was more than 95%. A low-dose CT scan was performed first for anatomical localization and attenuation correlation, and then PET imaging was conducted with 5-min acquisition.

### Image analysis

All images were analyzed by two experienced nuclear medicine physicians. For the semiquantitative analysis, the region of interest (ROI) was drawn at lung primary lesions on PET/CT images, and SUVmax was defined as the highest value in tumor burden. Meanwhile, average SUV (SUVmean) was defined as the mean value of the ROI. To avoid intrahepatic lesions, the SUVmean of the liver was calculated by a 30-mm-diameter ROI placing at the normal right hepatic lobe. To avoid partial-volume effects, the SUVmean of the blood pool was calculated by a 10-mm-diameter ROI placing at the middle of the descending aorta. SUR values were defined as the ratios of lung primary lesion SUVmax to liver and blood pool SUVmean (SUR-L and SUR-BP, respectively).

### Immunohistochemical staining

All tissues were fixed with 10% formalin for at least 6 h. All samples were embedded in paraffin and then hematoxylin–eosin staining (HE) and IHC were conducted. All samples were analyzed on an automated stainer with 22C3 (PD-L1 test kits, DAKO/Agilent, USA) (26). At least two pathologists evaluated the slides to determine the scores of PD-L1-positive cells on TC and IC. According to the FDA, EMA, and clinical trials (6, 7, 10, 11), we defined PD-L1 expression on TCs no less than 50% or ICs no less than 10% as positive. As for Ki-67, the high expression was defined as more than 20% (27).

### Statistical analysis

Statistical significance was defined as P < 0.05. Statistically significant differences were analyzed using chi-square test for categorical variables. Receiver operating characteristic (ROC) curve analyses were applied to test the continuous variables to discriminate negative and positive PD-L1 expression; the sensitivity and specificity were collected to determine the optimal cutoff value for continuous variables by ROC curves. The risk factors of PD-L1 expression were analyzed by univariate and multivariate analyses with logistic regression models. All statistical analyses were carried out with SPSS version 23.0 (SPSS Inc., Chicago, IL, USA).

## Results

### Patients’ characteristics

Clinicopathological features were summarized in **Table 1**. A total of 138 NSCLC patients were enrolled with 104 adenocarcinoma (ADC) and 34 squamous cell carcinoma (SCC). There were 36 patients (26%) expressing PD-L1 positively. In our NSCLC group, the positive expression was higher in patients who were men (33% *vs*. 15%, P = 0.026), with a smoking history (36% *vs*. 18%, P = 0.014), and with SCC (44% *vs*. 20%, P = 0.006). Ki-67 was stratified as low (≤20%) and high (>20%), and it was higher in the PD-L1-positive subset (60% *vs*. 15%, P = 0.000). The other characteristics showed no statistically significant difference between PD-L1-positive and PD-L1-negative patients, including age, maximum diameter, stage, and sample. While in ADC and SCC patients, the positive patients were 21 and 15, respectively. In ADC patients, the positive expression was linked to ages less than 64 years (29% *vs*. 10%, P = 0.026), smokers (33% *vs*. 13%, P = 0.022), and high Ki-67 (32% *vs*. 6%, P = 0.001). All of those characteristics had no correlation with PD-L1 in SCC patients.

**Table 1:** Characteristics of the PD-L1 expression.

	NSCLC				ADC				SCC			
Characteristics	PD-L1(-)	PD-L1(+)	χ^2^	P	PD-L1(-)	PD-L1(+)	χ^2^	P	PD-L1(-)	PD-L1(+)	χ^2^	P
Age (years)			0.479	0.489			5.736	0.026*			1.872	0.271
<64	47	19			39	16			8	3		
≥64	55	17			44	5			11	12		
Gender			4.957	0.026*			1.740	0.226			0.672	0.613
Men	58	28			42	14			16	14		
Women	44	8			41	7			3	1		
Diameter (mm)			0.403	0.525			1.494	0.326			2.524	0.139
<30	43	13			40	7			3	6		
≥30	59	23			43	14			16	9		
Smoking history			6.006	0.014*			6.110	0.022*			0.199	0.718
Smoker	41	23			27	13			14	10		
Non-smoker	61	13			56	8			5	5		
Stage			0.517	0.472			3.356	0.110			2.524	0.139
I–II	32	9			29	3			3	6		
III–IV	70	27			54	18			16	9		
Ki-67			14.643	0.000*			10.753	0.001*			0.672	0.613
Low	48	4			45	3			3	1		
High	54	32			38	18			16	14		
Histologic subtype			7.607	0.006*								
ADC	83	21										
SCC	19	15										
Histologic sample			1.679	0.432			3.679	0.188			1.440	0.258
Surgical	27	8			24	3			3	5		
Lesion biopsy	53	23			40	15			13	8		
Metastasis biopsy	22	5			19	3			3	2		

*P < 0.05; Ki-67: Low ≤20%, High >20%.

NSCLC, non-small cell lung cancer; ADC, adenocarcinoma; SCC, squamous cell carcinoma.

### ^18^F-FDG PET parameters

**Figure 1:**
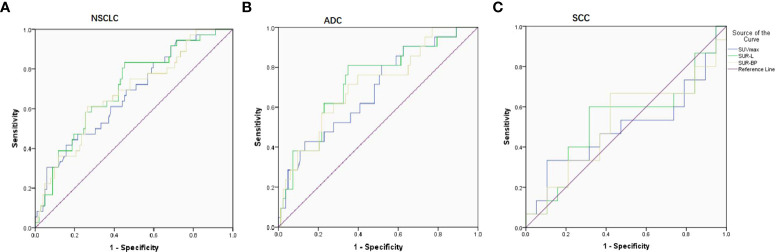
ROC curve on predicting PD-L1 expression based on three parameters (SUVmax, SUR-L, and SUR-BP). **(A)** In NSCLC patients, the cutoff value was 17.86, 4.84, and 8.98 with AUC being 0.671, 0.702, and 0.678, respectively. **(B)** In ADC patients, the cutoff value was 17.05, 4.96, and 7.30 with AUC being 0.682, 0.727, and 0.710, respectively. **(C)** In SCC patients, there were no differences with AUC being 0.498, 0.533, and 0.519.

The relationship between PET parameters and PD-L1 expression was shown in **Table 2** and **Figure 1**.

**Table 2:** The relationship between PET parameters and PD-L1 expression.

PD-L1 expression	PET parameters (mean ± SD)					
	NSCLC		ADC		SCC	
	Negative/Positive	P^#^	Negative/Positive	P^#^	Negative/Positive	P^#^
SUVmax	12.1±5.6/16.5±8.8	0.002	11.1±5.4/15.1±6.5	0.010	16.5±4.2/18.4±11.0	1.0
SUR-L	5.3±3.2/7.7±3.8	0.000	4.9±3.0/7.6±3.7	0.001	7.3±3.2/7.9±3.8	0.758
SUR-BP	7.8±5.4/11.1±6.0	0.001	7.3±5.4/11.1±5.9	0.003	10.2±4.5/11.2±6.2	0.864

^#^Mann–Whitney U test.

NSCLC, non-small cell lung cancer; ADC, adenocarcinoma; SCC, squamous cell carcinoma; SUVmax, the maximum of standard uptake value; SUR-L, ratio of lung lesion SUVmax to liver SUVmean; SUR-BP, ratio of lung lesion SUVmax to blood pool SUVmean.

In NSCLC patients, SUVmax (16.5 ± 8.8/12.1 ± 5.6, P = 0.002), SUR-L (7.7 ± 3.8/5.3 ± 3.2, P = 0.000), and SUR-BP (11.1 ± 6.0/7.8 ± 5.4, P = 0.001) were higher in the PD-L1-positive group. ROC determined the optimal SUVmax of 17.86 with an area under the curve (AUC) of 0.671 (P = 0.002). The sensitivity and specificity were 41.7% and 58.3%, respectively. The optimal SUR-L was 4.84 with a sensitivity of 83.3% and a specificity of 54.9% (AUC = 0.702, P = 0.000). The optimal SUR-BP was 8.98 with a sensitivity of 61.1% and a specificity of 73.5% (AUC = 0.678, P = 0.001).

In ADC patients, those parameters were higher in PD-L1-positive patients than those in PD-L1-negative patients, including SUVmax (15.1 ± 6.5/11.1 ± 5.4, P = 0.010), SUR-L (7.6 ± 3.7/4.9 ± 3.0, P = 0.001), and SUR-BP (11.1 ± 5.9/7.3 ± 5.4, P = 0.003). The best cutoff value of SUVmax, SUR-L, and SUR-BP determined by ROC was 17.05 (sensitivity = 42.9%, specificity = 82.7%, AUC = 0.682, P = 0.010), 4.96 (sensitivity = 81%, specificity = 65.1%, AUC = 0.727, P = 0.001), and 7.30 (sensitivity = 71.4%, specificity = 65.1%, AUC = 0.710, P = 0.003), respectively.

In SCC patients, there were no significant differences between PD-L1-positive and PD-L1-negative patients (SUVmax, AUC = 0.498, P = 0.986; SUR-L, AUC = 0.533, P = 0.742; SUR-BP, AUC = 0.519, P = 0.849).

**Figure 2:**
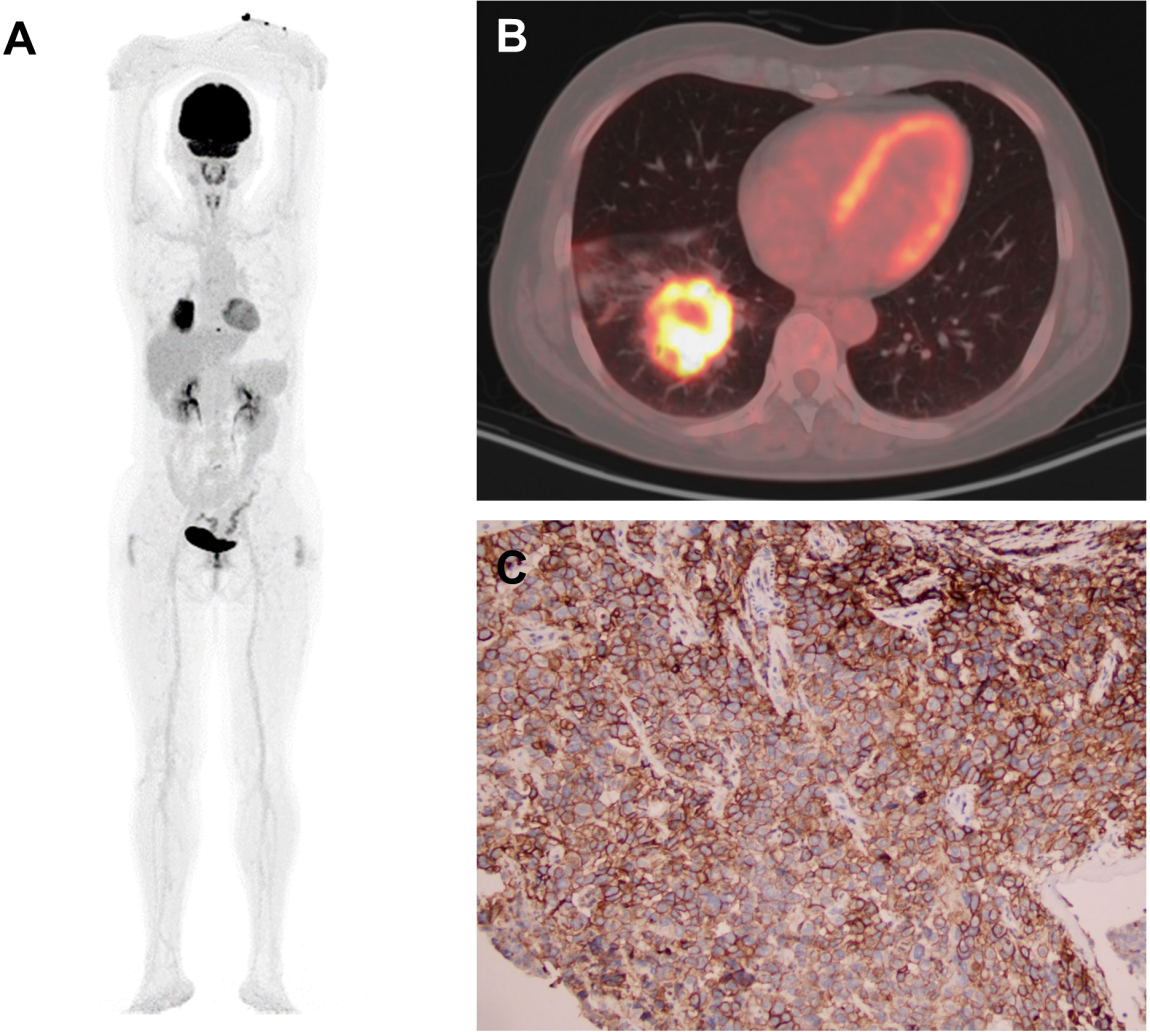
Representative of PET/CT and IHC staining: a 54-year-old female ADC patient. **(A, B)** PET images of MIP and CT with SUVmax 21.2, SUR-L 9.59, and SUR-BP 14.13. **(C)** Immunostaining image with PD-L1 expression on TCs 90% and ICs 30%. MIP, maximum intensity projection; CT, computed tomography; TC, tumor cell; IC, immune cell.

Representatives of total-body PET/CT and IHC staining were shown in **Figure 2**: a 54-year-old female ADC patient with SUVmax 21.2, SUR-L 9.59, and SUR-BP 14.13 and PD-L1 expression on TCs of 90% and ICs of 30%.

### Multivariate analysis of the relationship between programmed death ligand-1(PD-L1) expression and related variables

**Figure 3:**
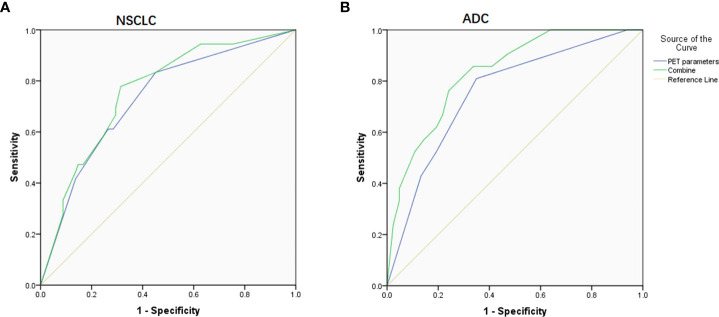
ROC curve on predicting PD-L1 expression based on PET parameters and combined them with clinical factors. **(A)** In NSCLC patients, the AUC was 0.730 and 0.758, respectively. **(B)** In ADC patients, the AUC was 0.756 and 0.833, respectively.

According to the obtained results, factors such as age, gender, smoking history, Ki-67, histologic subtype, SUVmax, SUR-L, and SUR-BP were included in the multivariate analysis, shown in **Table 3** and **Figure 3**. Multivariate analysis confirmed that Ki-67 was the only correlated factor in NSCLC patients. Age and SUR-L were correlated with PD-L1 expression in ADC patients.

**Table 3:** Multivariate analysis of the relationship between PD-L1 expression and related variables.

Factors	NSCLC		ADC	
	OR (95% CI)	P	OR (95% CI)	P
Age			0.203 (0.050-0.823)	0.026*
Gender	0.594 (0.154-2.289)	0.449		
Smoking	1.151 (0.355-3.737)	0.815	2.761 (0.867-8.792)	0.086
Ki-67	3.713 (1.126-12.240)	0.031*	2.784 (0.618-12.545)	0.183
Histologic subtype	1.360 (0.525-3.523)	0.527		
SUVmax	1.286 (0.408-4.048)	0.668	1.009 (0.226-4.511)	0.990
SUR-L	2.282 (0.642-8.109)	0.202	8.553 (1.170-62.552)	0.034*
SUR-BP	1.785 (0.517-6.164)	0.360	0.726 (0.114-4.620)	0.734

* P <0.05. OR: odds ratio; CI: confidence interval; SUVmax: the maximum of standard uptake value; SUR-L: the ratio of lung lesion SUVmax to liver SUVmean; SUR-BP: the ratio of lung lesion SUVmax to blood pool SUVmean.

Given that Ki-67 and histologic subtype could not be obtained by noninvasive tests, we then combined those available relevant factors including SUVmax, SUR-L, SUR-BP, age, gender, and smoking history and got ROC curves. When only the combined PET parameters were considered, the AUC was 0.730 and 0.756 for NSCLC and ADC, respectively. When gender, smoking history, and PET parameters were combined in NSCLC patients, the AUC was 0.758 (P = 0.000). When age, smoking history, and PET parameters were combined in ADC patients, the AUC was 0.833 (P = 0.000).

## Discussion

Immunotherapy with ICIs has prolonged the survival time of advanced NSCLC patients. Based on FDA and EMA, immunotherapy as first or second line is partly determined by the level of PD-L1 expression on TCs and/or ICs. Previous studies related to PET/CT mainly focused on SUVmax (22, 23), which can vary between different scanners (28). Therefore, we proposed SUR, which is a more stable and reliable PET parameter. Up to now, we were the first one to analyze the relationship between PD-L1 high expression and PET parameters obtained by the newest PET/CT machine.

In our study, there were 36 patients (26%) expressing PD-L1 positively. Previous studies got different results between PD-L1 expression on TCs and SUVmax in patients with NSCLC (22, 24, 29–33), ADC (23, 34, 35), and SCC (36, 37). In NSCLC patients, Zhao et al. (31) and Miyazawa et al. (32) concluded that SUVmax is linked to PD-L1 high expression on TCs. It was consistent with our research, although PD-L1 expression on ICs was also analyzed in our study. Hu et al. (22) comprehensively considered PD-L1 expression on both of TCs and ICs and got positive results. This study set 5% as the positive threshold. Although our positive value was higher, our result was in line with theirs. It could be explained by that PD-L1 expression is linked to hypoxia-inducible factor 1 alpha (HIF-1α) and glucose transporter 1 (Glut1) (34), and thus higher expression had higher SUVmax. In ADC patients, Hu et al. (23) had the same group as ours. They showed that stage was also related to the expression. However, in our research, age, smoking history, and Ki-67 were the other related factors. The study by Hu et al. (23) only included resected samples, but we enrolled I–IV patients. Maybe different samples could explain that. In SCC patients, Zhang et al. (36) and Kasahara et al. (37) found that SUVmax is positively related to the expression, regardless of setting the positive threshold as 5% or 26%. Our result did not support it. After all, we included the expression on ICs, but they did not. The different positive threshold and test antibodies were also part of the reasons.

SUVmax has high reproducibility, but it can vary based on different scanners, institutional parameters, and patients’ metabolism (20, 28). To overcome the potential variability of SUVmax, tumor SUV is normalized by liver and blood pool activity, which can be more reliable and stable (21, 38). As our results showed, in NSCLC and ADC patients, SUR-L and SUR-BP were higher in the PD-L1-positive subset. Multivariate analysis confirmed that Ki-67 was the only correlated factor in NSCLC patients. In ADC patients, age and SUR-L were related factors. As we know, Ki-67 and histologic subtype cannot be acquired from invasive tests. Therefore, we conducted multivariate analysis excluding them. We concluded that SUR-L was the only correlated factor in NSCLC, and age, smoking history, and SUR-L were relevant factors in ADC (not shown). In addition, the AUC on ROC curve for SUR was higher than that for SUVmax. Therefore, we got a conclusion that SUR was better than SUVmax in predicting PD-L1 expression. Our result also found that SUR had higher sensitivity and SUVmax had higher specificity. When PET parameters were combined, it had improvement in predicting PD-L1 expression. Then, we combined PET parameters and clinical factors, and it presented a better predictive efficacy in distinguishing PD-L1 expression, especially in ADC patients, with an AUC being 0.833.

As we mentioned above, all of the patients were scanned by total-body PET/CT (uEXPLORER), and Tan et al. (39) had pointed out that the new machine with half-dose ^18^F-FDG can achieve better image quality than conventional PET/CT with full dose. In their study, they demonstrated that SUR was almost the same between the two machines in the same patient. Thus, SUR has a higher reference value. In NSCLC and ADC patients of our study, the AUC and Youden Index of SUR-L were bigger than those of SUR-BP, which meant that SUR-L could be a better index than SUR-BP. Moreover, the standard deviation (SD) of SUR-L was smallest compared with the other two. Theoretically, our study confirmed that SUR-L could be a more reliable and stable parameter in predicting PD-L1 high expression.

The newest total-body PET/CT can achieve lower injection dose, faster scanning speed, and higher resolution than the others (15, 40, 41). Therefore, it is more valuable in clinical application. In our study, we were the first one to explore its value on immunotherapy in NSCLC patients. Previous studies point out that PD-L1 expression on TCs is related to SUVmax (22–25). The NSCLC issues include TCs and ICs and both of them can express PD-L1 (6, 8). Based on that, we defined PD-L1 high expression. Our results demonstrated that PD-L1 high expression was associated with SUVmax, SUR-L, and SUR-BP. When they were combined with clinical factors, the accuracy could be higher. Moreover, a prospective clinical trial (NCT04654234) was recently launched to explore the value of the newest total-body PET/CT in accessing prognosis after neoadjuvant chemotherapy plus nivolumab in stage III NSCLC patients. Our result might provide some references for this clinical trial, and our predictive value of the high expression might be verified.

There were several limitations in our study. First, although we adopted three metabolic parameters, PET/CT did not show a prognostic value in patients after immunotherapy. However, some studies had found that SUVmax can be a prognostic biomarker in patients treated with ICIs (42–44). SUR also has prognostic value in patients with lung cancer (16, 19). However, it still needs more research to explore the relationship between SUR and survival time after immunotherapy. Second, we did not include other receptors, such as Epidermal Growth Factor Receptor (EGFR) and Anaplastic Lymphoma Kinase (ALK). So, our result only offers related information for patients who will receive first-line atezolizumab, and a more detailed research is needed. Third, other metabolic parameters, such as metabolic tumor volume (MTV) and total lesion glycolysis (TLG) (35, 42), also have certain value.

## Conclusion

In conclusion, SUVmax, SUR-L, and SUR-BP had utility in predicting PD-L1 high expression, and SUR-L was the most reliable value. In the present study, we found a more reliable PET parameter to predict PD-L1 high expression and increased the accuracy of the expression by combining PET parameters and clinical factors, which could provide reference for clinicians to screen patients who could be more suitable for first-line atezolizumab therapy.

## Data availability statement

The original contributions presented in the study are included in the article/supplementary material. Further inquiries can be directed to the corresponding authors.

## Ethics statement

This study was reviewed and approved by the ethics committee of Henan Provincial People’s Hospital, Zhengzhou University, Zhengzhou, Henan, China. The patients/participants provided their written informed consent to participate in this study.

## Author contributions

BH collected, analyzed data and wrote the manuscript. HJ made efforts to collect data. XL analyzed the data. XW controlled the study patients. JX and YG, as the corresponding authors, made the major contributions to design, conduct the study and revise the manuscript. All authors contributed to the article and approved the submitted version.

## Funding

This work was supported by the Fund program of Science and Technology Research of Henan Provincial Health and Health Commission (SBGJ202102015).

## Conflict of interest

The authors declare that the research was conducted in the absence of any commercial or financial relationships that could be construed as a potential conflict of interest.

## Publisher’s note

All claims expressed in this article are solely those of the authors and do not necessarily represent those of their affiliated organizations, or those of the publisher, the editors and the reviewers. Any product that may be evaluated in this article, or claim that may be made by its manufacturer, is not guaranteed or endorsed by the publisher.
